# Impact of Preoperative Nutritional Support on Surgical Outcomes in Gastrointestinal Surgeries: A Systematic Review

**DOI:** 10.7759/cureus.56416

**Published:** 2024-03-18

**Authors:** Muhammad Muaz Loon, Mulusew Goshe, Muhammad Rashid, Abdullah Shehryar, Abdur Rehman, Shenouda Abdallah, Shariq K Baluch, Arslan Ahmed, Hanen Batat, Maria Quinn

**Affiliations:** 1 Surgery, Mayo Hospital, Lahore, PAK; 2 Medicine, Jimma University, Jimma, ETH; 3 Orthopedic Surgery, Addis Ababa University, Addis Ababa, ETH; 4 General Surgery, Allama Iqbal Medical College, Lahore, PAK; 5 Internal Medicine, Allama Iqbal Medical College, Lahore, PAK; 6 Surgery, Jaber Al-Ahmad Hospital, Kuwait, KWT; 7 Internal Medicine, Universidad Autonoma de Guadalajara, Guadalajara, MEX; 8 Internal Medicine, King Edward Medical University, Lahore, PAK; 9 Faculty of Medicine, Yarmouk University, Irbid, JOR; 10 Internal Medicine, Jinnah Hospital Lahore, Lahore, PAK

**Keywords:** recovery, postoperative complications, surgical outcomes, gastrointestinal surgery, preoperative nutritional support

## Abstract

Malnutrition presents a significant risk to patients undergoing gastrointestinal surgery, with direct consequences on postoperative complication rates, recovery times, and mortality. Our systematic review, guided by PRISMA protocols, examined the impact of preoperative nutritional support on these surgical outcomes. We scrutinized publications from PubMed, Medline, Embase, and the Cochrane Library up to April 2023, including randomized controlled trials, cohort studies, and systematic reviews. The stringent selection process narrowed to 10 studies demonstrating the efficacy of preoperative nutritional support, from oral supplements to enteral and parenteral nutrition, in reducing postoperative complications and length of hospital stays while enhancing recovery rates. The benefits varied, indicating a pressing need for customized nutritional regimens based on patient demographics and surgical specifics. Our findings advocate incorporating individualized nutritional strategies into preoperative care, enhancing patient outcomes. Future research should aim to refine these strategies, focusing on the optimal timing, duration, and type of nutritional support.

## Introduction and background

Malnutrition is a prevalent concern among patients undergoing gastrointestinal surgeries, significantly impacting surgical outcomes. Studies have demonstrated that malnourished patients are at a higher risk of postoperative complications, longer hospital stays, and increased mortality rates. In this context, preoperative nutritional support is crucial to improving surgical outcomes and patient recovery. The role of nutritional support, encompassing oral supplements, enteral, and parenteral nutrition, in enhancing immune function, reducing the risk of infections, and promoting wound healing is well-documented in the medical literature [1].

Recent advancements in clinical nutrition have underscored the importance of tailored nutritional interventions in the preoperative period. Immune-modulating diets, rich in arginine, omega-3 fatty acids, and nucleotides, have shown promise in reducing postoperative complications and improving recovery rates. Moreover, the timing and duration of nutritional support have emerged as pivotal factors in optimizing surgical outcomes, highlighting the need for a nuanced understanding of preoperative nutritional strategies [2].

Despite the growing body of evidence supporting the benefits of preoperative nutritional support, there remains a gap in comprehensive reviews synthesizing this knowledge, particularly focusing on gastrointestinal surgeries. This gap is notable against evolving clinical practices and the emergence of new research findings. Consequently, healthcare professionals and policymakers require up-to-date, evidence-based insights to inform clinical guidelines and improve patient care standards in surgical settings [3].

Incorporating insights from recent studies, the efficacy of specific nutritional supplements has become increasingly evident in the context of preoperative care. For instance, research by Wada et al. explored the use of a nutritional supplement containing beta-hydroxy-beta-methylbutyrate, arginine, and glutamine (HMB/Arg/Gln), which, although not significantly reducing wound complications, indicated an increase in serum growth hormone levels within the treatment group. This suggests potential benefits that warrant further investigation [4]. Moreover, a systematic review and meta-analysis by Chen et al. highlighted the significant role of immune-modulating nutrition (IMN) in reducing hospital stays and infectious complications in patients undergoing pancreaticoduodenectomy (PD). These findings underscore the importance of IMN, particularly in specific patient groups, thus advocating for its inclusion in preoperative nutritional strategies [5]. These studies reinforce the critical role of tailored nutritional interventions, including specific nutrient formulations, in enhancing surgical outcomes, further emphasizing the need for personalized approaches in preoperative nutritional care.

The primary objective of our systematic review is to critically evaluate and synthesize the existing literature on the impact of preoperative nutritional support on surgical outcomes in patients undergoing gastrointestinal surgeries. We aim to explore the relationship between different forms of nutritional support (oral supplements, enteral, and parenteral nutrition) and their timing and duration relative to surgery, with surgical outcomes such as postoperative complications, length of hospital stays, recovery rate, wound healing, infection rates, and overall patient mortality and morbidity. This comprehensive synthesis will highlight the current state of evidence, identify knowledge gaps, and suggest future research directions. Through our systematic review, we strive to provide a robust foundation for developing clinical guidelines that optimize preoperative nutritional interventions, ultimately improving surgical outcomes and patient quality of life in the context of gastrointestinal surgeries.

## Review

Materials and methods

Search Strategy

Our search strategy, developed in line with the PRISMA guidelines, aimed to systematically identify all relevant studies examining the impact of preoperative nutritional support on surgical outcomes in gastrointestinal surgeries. We conducted extensive searches across several electronic databases, including PubMed, Medline, Embase, and the Cochrane Library, from their inception until April 2023.

The search strategy utilized a combination of keywords and MeSH terms specifically related to our research question. Keywords such as “preoperative nutritional support,” “gastrointestinal surgery outcomes,” “perioperative nutrition,” and “surgical recovery” were used alongside MeSH terms, including “Preoperative Care,” “Nutritional Support,” “Gastrointestinal Surgical Procedures,” and “Postoperative Complications.” Boolean operators “AND” and “OR” facilitated an effective combination of these terms, for example, “preoperative nutritional support AND gastrointestinal surgery outcomes” or “perioperative nutrition OR surgical recovery.”

To ensure a comprehensive literature search, we also examined the reference lists of selected articles for additional relevant studies. Our search scope was broadened to include clinical trial registries and conference proceedings, capturing unpublished or ongoing studies that could be pertinent to our review. An expert in the field of medical information retrieval critically evaluated the search strategy to confirm its alignment with the PRISMA guidelines and its comprehensiveness in covering the topic of preoperative nutritional support in gastrointestinal surgeries.

Eligibility Criteria

We have established specific eligibility criteria to ensure the integrity and focus of our systematic review. The inclusion criteria target peer-reviewed research articles, randomized controlled trials (RCTs), cohort studies, systematic reviews, and meta-analyses that investigate the impact of preoperative nutritional support on surgical outcomes in gastrointestinal surgeries. We are particularly interested in studies that evaluate outcomes such as postoperative complications, length of hospital stay, recovery rate, wound healing, infection rates, and overall patient mortality and morbidity. This review also considers studies examining various forms of nutritional support, including oral supplements, enteral nutrition, and parenteral nutrition, and their timing and duration relative to surgery.

Only studies conducted on adult human populations undergoing gastrointestinal surgeries are included to ensure the applicability of the results to the intended patient group. To maintain linguistic accessibility and the feasibility of a thorough review, we restrict our inclusion to English-language studies.

Conversely, the exclusion criteria are designed to narrow the focus of our review to the most relevant and high-quality studies. Our review excludes studies not directly assessing the impact of preoperative nutritional support on gastrointestinal surgical outcomes. Studies focusing solely on pediatric populations, animal models, or non-surgical interventions are excluded to maintain a clear focus on adult surgical outcomes. Additionally, non-English language studies and grey literature, including conference abstracts, posters, unpublished works, and reports with insufficient data on the outcomes of interest, are also excluded. This exclusion criterion ensures that our review is based on reliable, comprehensive, high-quality scientific evidence that addresses our research question. This structured approach to defining eligibility criteria aims to capture the most pertinent and high-quality studies, contributing significantly to understanding preoperative nutritional support's role in improving gastrointestinal surgical outcomes.

Data Extraction

In our systematic review, data extraction was executed precisely to guarantee the accuracy and relevance of information from the studies. Initially, titles and abstracts were screened by two independent reviewers to discern studies that aligned with our objectives and categorized as relevant, potentially relevant, or not relevant. Full-text articles classified as potentially relevant underwent a rigorous appraisal. Using a standardized Excel template, the reviewers systematically extracted data in line with predetermined criteria, ensuring uniformity and meticulousness in the process. Discrepancies were resolved through consultation with a third reviewer, ensuring consensus and the review's integrity.

The extraction template was specifically designed to capture critical data, including publication details, study demographics, and intricate aspects of preoperative nutritional support type, duration, and timing, which are vital for appraising their impact on surgical outcomes. Study design, setting, and outcomes, such as postoperative complications and recovery rates, were also documented, facilitating a comprehensive assessment of the studies' quality and applicability.

This detailed and structured extraction process allowed for an in-depth and accurate analysis of the influence of preoperative nutritional support on gastrointestinal surgery outcomes, enhancing the scholarly rigor of our synthesis. The methodology ensures a transparent and thorough evaluation, reinforcing the importance of nutritional support in preoperative care for these patients.

Data Analysis and Synthesis

Our systematic review adopted a narrative synthesis for data analysis to intricately understand the role of preoperative nutritional support in gastrointestinal surgeries. We identified key patterns and themes by summarizing and categorizing findings from diverse studies- considering surgical types, nutritional interventions, and patient demographics. These included the efficacy of different nutritional supports, their timing and duration, and patient adherence, all critical in shaping surgical outcomes and recovery.

Through thematic and comparative analysis, we discerned the varied impact of nutritional strategies and recognized areas where evidence is robust or lacking. This facilitated a critical evaluation within the broader context of existing research and clinical practice, informing implications for patient outcomes and guidelines. The synthesis culminated in conclusions and recommendations, underscoring the importance of tailored preoperative nutritional support and pinpointing directions for future research to refine clinical practices.

Results

Study Selection Process

The systematic search strategy employed across various databases and registers resulted in the identification of 1,032 records. The initial phase of our study selection involved removing duplicates, which in this case accounted for 165 records, subsequently refining the total to 867 records for title and abstract screening. This screening process further narrowed the field, excluding 351 records and leaving 516 reports for full-text retrieval.

However, a substantial number of these reports, 394 in total, were not retrieved, which led to an in-depth assessment of the remaining 122 reports for eligibility. This assessment was based on strict inclusion and exclusion criteria that had been predetermined to ensure the relevance and quality of the studies. Through this rigorous evaluation, 112 reports were excluded, resulting in a concise number of 10 studies deemed suitable for inclusion in the review.

**Figure 1:**
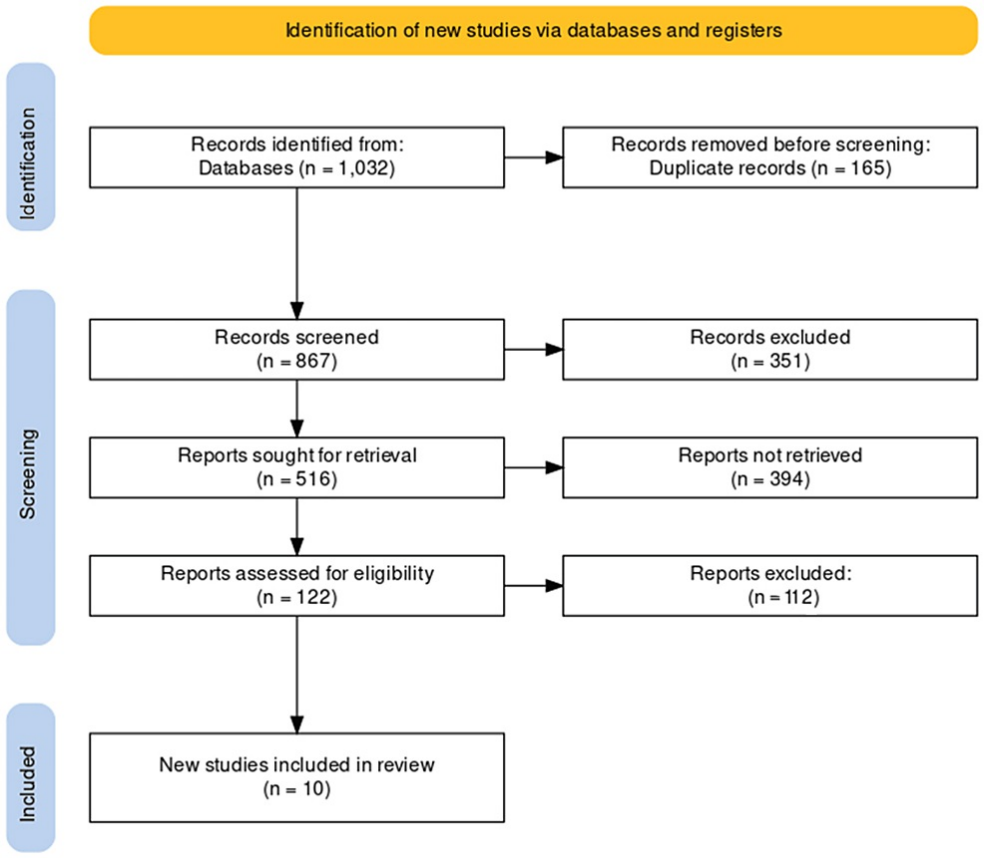
PRISMA flow diagram of the selection of studies for inclusion in the systematic review.

As delineated in the PRISMA flowchart in Figure 1, this meticulous process ensures a comprehensive and unbiased selection of studies while providing a transparent and systematic visual representation of the methodology applied during the study selection phase.

Characteristics of Selected Studies

Our systematic review encompasses a detailed analysis of ten pivotal studies, each contributing valuable insights into the effects of preoperative nutritional support on outcomes in gastrointestinal surgeries. These selected studies encompass a range of research designs, including RCTs, systematic reviews and meta-analyses, and observational studies, reflecting a broad spectrum of clinical inquiries and outcomes. The studies cover diverse patient populations undergoing various types of gastrointestinal surgeries, with sample sizes ranging from 40 to 907 participants.

Key aspects of each study, such as the lead co-author, publication year, study objectives, design, population, sample size, main findings, and conclusions, are briefly summarized. This summary provides a clear overview of the scope, methodology, and implications of the research conducted on preoperative nutritional support in this field. Table 1 serves as an essential reference, offering a snapshot of each study's critical characteristics and contributions to facilitate a comprehensive understanding of the current evidence base regarding nutritional support in the preoperative setting for gastrointestinal surgery patients.

**Table 1:** Summary of key studies on the impact of preoperative nutritional and supportive interventions on surgical outcomes in gastrointestinal surgeries. RCT: Randomized Controlled Trial, IMN: Individualized Nutritional Management, PD: Parkinson's Disease, DGE: Delayed Gastric Emptying, ICU: Intensive Care Unit, PPCs: Postoperative Pulmonary Complications

Study	Lead Co-Author	Year	Objective	Study Design	Population	Sample Size	Main Findings	Conclusion
1	Noriko Wada [4]	2018	Evaluate perioperative nutrition using HMB/Arg/Gln	RCT	Abdominal malignancies surgery	60	No reduction in wound complications; increased serum GH in HMB/Arg/Gln group	HMB/Arg/Gln didn't reduce wound complications; further research is needed
2	Yan Chen [5]	2018	Effects of IMN on PD patients	Systematic Review & Meta-analysis	PD patients	572	IMN reduced hospital stays and infectious complications	IMN is recommended for PD; more research is needed
3	Fabio Ausania [6]	2019	Impact of prehabilitation on PD complications	RCT	PD candidates	40	No difference in overall/major complications; reduced DGE in the prehabilitation group	Prehabilitation didn't reduce complications but reduced DGE
4	Da-Li Sun [7]	2017	Efficacy of multi-modal EON program	Randomized Trial	Major abdominal surgery	107	Improved oral nutrition tolerance, reduced stay and costs	Multi-modal EON improves outcomes; further research on applications needed
5	X Sun [8]	1998	Cholesterol concentrations post-surgery	Observational Study	Postsurgical ICU patients	213	Significant decrease in cholesterol and proteins post-surgery	Cholesterol not reliable for nutritional assessment post-surgery
6	Hairil Rizal Abdullah [9]	2017	Prehabilitation bundle in frail elderly	Randomized Trial	Frail elderly, abdominal surgery	62	Primary outcome: length of stay. Results pending	The study assesses prehabilitation's effect on surgery recovery
7	Ianthe Boden [10]	2018	Preoperative physiotherapy on PPCs	Randomized Trial	Elective upper abdominal surgery	441	Halved incidence of PPCs in the intervention group	Preoperative physiotherapy significantly reduces PPCs
8	Michael J Hughes [11]	2019	Effect of prehabilitation on surgery outcomes	Systematic Review & Meta-analysis	Prehabilitation before abdominal surgery	907	Reduced overall and pulmonary morbidity	Prehabilitation should be routine; optimal protocol is needed
9	Toby Richards [12]	2020	Preoperative intravenous iron on anemia	Randomized Trial	Anemic patient's abdominal surgery	487	No difference in transfusion needs or outcomes	Preoperative iron is not superior to a placebo for reducing transfusions
10	Jeremy Meyer [13]	2022	Pre-operative iron on hemoglobin concentration	Systematic Review & Meta-analysis	Major abdominal surgery	4 RCTs	Increased hemoglobin concentration; no reduction in transfusions	Increases hemoglobin but doesn't reduce transfusion need; more targeted trials needed

Discussion

In synthesizing the findings from the ten pivotal studies in our systematic review on the impact of preoperative nutritional support on surgical outcomes in gastrointestinal surgeries, several key insights emerge. Wada et al. [4] explored the use of HMB/Arg/Gln supplementation, finding no significant reduction in wound complications, although there was an increase in serum growth hormone levels within the treatment group, suggesting potential areas for further research. Similarly, Chen et al. [5] reported that IMN significantly reduced hospital stay and infectious complications in patients with PD, underscoring the benefits of IMN in this specific patient population.

Ausania et al. [6] examined the impact of prehabilitation on PD complications, revealing no significant difference in overall or major complications, yet noting a reduction in delayed gastric emptying (DGE), indicating a nuanced benefit of prehabilitation. The study by Sun et al. [7] highlighted the efficacy of a multi-modal early oral nutrition (EON) program in improving oral nutrition tolerance and reducing hospital stay and costs, suggesting the value of EON programs in major abdominal surgery recovery.

Sun's [8] observational study on cholesterol concentrations post-surgery in postsurgical ICU patients found a significant decrease in cholesterol and proteins, questioning the reliability of cholesterol as a nutritional assessment marker post-surgery. Abdullah [9] focused on a prehabilitation bundle in frail elderly patients undergoing abdominal surgery, with primary outcomes including the length of stay still pending, highlighting the importance of prehabilitation in this vulnerable population.

Boden [10] demonstrated that preoperative physiotherapy significantly reduced the incidence of postoperative pulmonary complications (PPCs) in patients undergoing elective upper abdominal surgery, advocating for the routine inclusion of physiotherapy in preoperative care. Hughes [11] and Richards [12] further emphasized the importance of prehabilitation. Hughes suggested reduced overall and pulmonary morbidity, and Richards found no significant difference in the need for transfusions or outcomes with preoperative intravenous iron, indicating the need for more targeted trials.

Lastly, Meyer's [13] systematic review and meta-analysis on pre-operative iron supplementation showed increased hemoglobin concentration but no reduction in transfusion needs, pointing toward the necessity for more focused research in this area.

These studies underscore the complex and multifaceted impact of preoperative nutritional and supportive interventions on surgical outcomes in gastrointestinal surgeries. While certain interventions like IMN and prehabilitation promise to improve patient outcomes, the variability in effectiveness across different measures and patient populations highlights the need for further, more targeted research to optimize preoperative care protocols​​​​.

Contextualizing the findings of our systematic review with existing literature reveals a nuanced landscape of preoperative nutritional support's role in gastrointestinal surgeries. The utility of specific nutritional supplements, such as those containing HMB/Arg/Gln, as explored by Wada et al. [4], aligns with the broader discourse on the potential of amino acid supplementation to enhance surgical recovery. However, contrasting results from studies like Gustafsson et al. [14], which found preoperative nutritional support to significantly reduce postoperative complications in colorectal surgery patients, suggest that the impact of nutritional interventions may vary by surgical type and patient population.

The effectiveness of IMN in reducing hospital stay and infectious complications, as reported by Chen et al. [5], echoes findings from Drover et al. [15], who highlighted the role of IMN in improving outcomes for patients undergoing major gastrointestinal surgery. These studies collectively affirm the critical role of tailored nutritional strategies in surgical care, albeit with variations in specific outcomes and patient benefits.

The concept of prehabilitation, including nutritional, physical, and psychological preparations before surgery, has gained traction. The work by Ausania et al. [6] and the broader implications of prehabilitation strategies underscored by Hughes [11] resonate with the findings from Gillis et al. [16], who advocate for multimodal prehabilitation to enhance postoperative recovery. This body of work collectively supports the integration of prehabilitation into standard preoperative care, though it also calls for further research to delineate optimal protocols.

Moreover, as discussed by Richards [12] and Meyer [13], the limited impact of preoperative iron supplementation on reducing transfusion needs presents an intriguing contrast to earlier optimism about iron's role in improving perioperative anemia and surgical outcomes. This discrepancy suggests a more complex interaction between iron status and surgical recovery than previously understood, as explored in studies like Beris et al. [17], highlighting the potential benefits of correcting preoperative anemia with iron supplementation.

Our review adds to the existing knowledge by providing a comprehensive synthesis of recent evidence on preoperative nutritional support in gastrointestinal surgeries. It reinforces the importance of nutritional interventions in improving surgical outcomes and highlights the variability in effectiveness and the need for personalized approaches. These findings underscore the ongoing evolution of preoperative care practices and the necessity for continued research to refine and optimize nutritional support strategies for surgical patients [18,19].

Preoperative nutritional support influences surgical outcomes through various physiological and biochemical mechanisms. Nutritional interventions, particularly those providing immune-modulating nutrients such as arginine, omega-3 fatty acids, and nucleotides, can enhance immune function, modulate the body's inflammatory response, and support the maintenance of gut integrity. This immune enhancement is crucial for reducing postoperative infection rates and improving wound healing. For instance, arginine is known to support nitric oxide production, which plays a significant role in wound healing and immune function. Similarly, omega-3 fatty acids can alter cell membrane compositions, influencing inflammatory response pathways and potentially reducing the incidence of postoperative complications [20]. Furthermore, adequate preoperative nutrition helps maintain muscle mass and strength, reducing the risk of post-surgical complications such as infections and prolonged hospital stays. Collectively, these mechanisms underscore the multifaceted role of nutritional support in optimizing patient outcomes in gastrointestinal surgeries, highlighting the importance of targeted nutritional strategies to address specific patient needs and surgical demands [21].

The systematic review's strengths lie in its rigorous methodology, adherence to PRISMA guidelines, and comprehensive search strategy, ensuring wide coverage of relevant literature on preoperative nutritional support in gastrointestinal surgeries [22]. Including diverse study designs enriches the review's findings, offering a broad perspective. However, the review faces limitations, including potential publication bias, the heterogeneity of interventions, and variability in study quality. These factors may affect the generalizability of the findings. The diversity in nutritional interventions and patient populations also introduces challenges in directly comparing outcomes, underscoring the need for a cautious interpretation of the results.

Our review underscores the critical role of preoperative nutritional support in improving outcomes for gastrointestinal surgery patients. Timely and tailored nutritional strategies, essential for optimizing surgical results, should factor in costs, patient adherence, and available healthcare resources. A multidisciplinary approach is necessary for effective preoperative care planning.

Further research is needed to address the gaps in evidence through more comprehensive RCTs, particularly regarding the impact of various supplements and the fine-tuning of administration protocols. Studying specific demographics like the elderly or malnourished and assessing long-term recovery impacts are also crucial. Our analysis indicates that individualized nutritional plans, especially those with immune-modulating nutrients, can significantly reduce complications and expedite recovery. This review advocates incorporating such nutritional support into clinical practice, enhancing patient care, and advancing surgical outcomes.

## Conclusions

Our systematic review elucidates the pivotal role of preoperative nutritional support in enhancing outcomes for gastrointestinal surgery patients, emphasizing its necessity for an optimal preoperative care regimen. The synthesis of current evidence reveals that strategic nutritional interventions can significantly mitigate postoperative complications, expedite recovery processes, and improve overall patient well-being. While the review identifies promising areas for the application of nutritional support, it also highlights the need for further research to refine these strategies and tailor them to individual patient profiles. Ultimately, incorporating comprehensive nutritional assessments and interventions into preoperative planning stands as a critical advancement in surgical care, promising to elevate patient outcomes and set new standards in healthcare efficacy and patient-centered care.
